# Potential Biomarkers and Treatment of Neuroinflammation in Parkinson’s Disease

**DOI:** 10.62641/aep.v53i1.1779

**Published:** 2025-01-05

**Authors:** Ziqi Zhao, Qiang Fu, Xiangyu Guo, Huihan He, Ge Yang

**Affiliations:** ^1^College of Traditional Chinese Medicine, Changchun University of Traditional Chinese Medicine, 130117 Changchun, Jilin, China; ^2^Department of Geriatrics, Affiliated Hospital of Changchun University of Traditional Chinese Medicine, 130021 Changchun, Jilin, China

**Keywords:** Parkinson's disease, neuroinflammation, inflammatory factors, biomarkers, heal

## Abstract

Parkinson's disease (PD) is a degenerative disease of the central nervous system primarily affecting middle-aged and elderly individuals, significantly compromising their quality of life. Neuroinflammation is now recognized as a key feature in the pathogenesis of PD. This study reviews recent advances in the identification of potential biomarkers associated with neuroinflammation in PD and their significance for therapeutic strategies. These findings suggest that inflammatory factors play a pivotal role in PD treatment, and interventions involving anti-inflammatory drugs, physical exercise, and dietary modifications have shown promising results in mitigating disease progression.

## Introduction

Parkinson’s disease (PD), also known as paralysis tremor, is a neurodegenerative disorder primarily characterized by slow movement, rigidity, and tremor. In addition to motor symptoms, PD patients frequently experience non-motor symptoms, including anosmia, constipation, urinary dysfunction, orthostatic hypotension, memory loss, depression, pain, and sleep disorders. Cognitive dysfunction, in particular, significantly diminishes the quality of life for those affected [1, 2, 3].

The prevalence of PD is increasing globally. A national community-based study in China reported that 1.37% of individuals over 60 years of age were affected by PD, translating to an estimated 3.62 million patients [4]. Similarly, a German study found that the age-standardized prevalence of PD ranged from 797 to 961 per 100,000 people [5]. These data underscore the substantial and growing burden of Parkinson’s disease, which is expected to rise further as populations continue to age.

Despite the extensive research, the exact etiology of PD remains unclear. Current understanding suggests that the primary pathological features of PD include the degeneration and death of dopaminergic neurons in the substantia nigra, and the formation of Lewy bodies in the brain. These changes result in a significant decline in dopamine (DA) levels in the striatum, leading to the neurological symptoms observed in patients. However, the precise mechanisms underlying these processes are not fully understood [6].

Neuroinflammation is considered a critical pathological feature throughout the progression of PD. The activation of microglia and astrocytes and the subsequent release of cytokines and reactive oxygen species (ROS) contribute to neuroinflammation in the nervous system, disrupting the blood-brain barrier and exacerbating DA neuron death [7]. A deep understanding of neuroinflammation in Parkinson’s disease is essential for developing effective treatments. In this review, we summarize recent advances in identifying markers associated with PD-related neuroinflammation and explore their potential implications for clinical management.

## The Role of Neuroinflammation in PD

The blood-brain barrier (BBB) is designed to separate the central nervous system (CNS) from the peripheral circulation, which has historically led to the belief that the CNS is an immune-privileged area. However, increasing evidence suggests that the CNS can exhibit inflammatory responses to injury or infection [8]. Neuroinflammation, typically a chronic rather than an acute response, is a protective mechanism within the CNS. However, neuroinflammation can have protective and harmful effects depending on its intensity and duration. Excessive secretion of inflammatory mediators is generally believed to cause damage to the CNS, contributing to various neurodegenerative diseases through prolonged neural injury [9].

In Alzheimer’s disease (AD), for instance, elevated levels of the neuroinflammatory marker Glial fibrillary acidic protein were observed in preclinical subjects compared to healthy controls, highlighting the role of neuroinflammation in AD progression [10]. Similarly, research by Cui *et al*. [11] demonstrated that the polyunsaturated fatty acid (PUFA) metabolic enzyme acyl-CoA synthetase long-chain family member 4 (ACSL4) can exacerbate ischemic stroke by promoting ferroptosis-induced brain damage and neuroinflammation.

Neuroinflammation plays a particularly prominent role in PD. As early as 1988, McGeer *et al*. [12] identified abundant reactive microglia in the substantia nigra of PD patients, marking the beginning of PD-related neuroinflammation research. Subsequent study revealed an increased number of major histocompatibility complex (MHC) II-positive cells in the brain of PD patients compared to healthy individuals [13]. Recent advancements in brain imaging technologies have further strengthened the relationship between neuroinflammation and PD. A systematic review and meta-analysis by Peng-Fei Zhang and Fan Gao [14] utilized positron emission tomography (PET) to assess translocator protein (TSPO) levels, an indicator of neuroinflammation, in PD patients. The analysis, which included 15 studies with 455 participants, found elevated TSPO levels in multiple brain regions of PD patients, including the midbrain, putamen, anterior cingulate gyrus, posterior cingulate gyrus, thalamus, striatum, and frontal and temporal lobes [14]. These findings underscore the close relationship between neuroinflammation and PD.

PD-related neuroinflammation is closely related to microglia. Microglia constitute the resident cell population of the human central nervous system, which is derived from red myeloid progenitor cells and depends on transcription factor PU.1 and interferon (IFN) regulatory factor 8 (IRF8) signaling. Under normal conditions, microglia maintain homeostasis through slow proliferation and long life spans. However, in response to a disease, they rapidly proliferate and adopt an activated state [15, 16]. In the CNS, microglia interact with neurons to mediate phagocytosis, apoptosis, and the removal of non-functional synapses, thereby preserving neuronal networks and brain health [17]. In their resting state, microglia exhibit a steady-state phenotype, but when triggered by brain injury or other disturbances, they shift to a reactive state, adopting a pro-inflammatory M1 phenotype. This shift results in the production of ROS and pro-inflammatory cytokines, such as interleukin (IL)-6, IL-1β, nitric oxide synthase (NOS), and tumor necrosis factor α (TNF-α), which contribute to neuronal degeneration and progression of PD [18, 19].

Astrocytes, the most abundant glial cells in the CNS, also play a crucial role in PD-related neuroinflammation. These cells regulate synaptic and neuronal activities, maintain the integrity of the BBB, and ensure proper cerebral blood flow [20, 21]. Under pathological conditions and inflammatory reactions, astrocytes interact with microglia to amplify immune responses and activate apoptotic pathways, leading to dopaminergic neuron (Dan) death. As inflammatory mediators, astrocytes produce cytokines such as IL-1, IL-5, IL-6, TNF-α, TGF-β, IL-1α, and IL-1β in the brain, further driving PD progression [22].

**Fig. 1.**
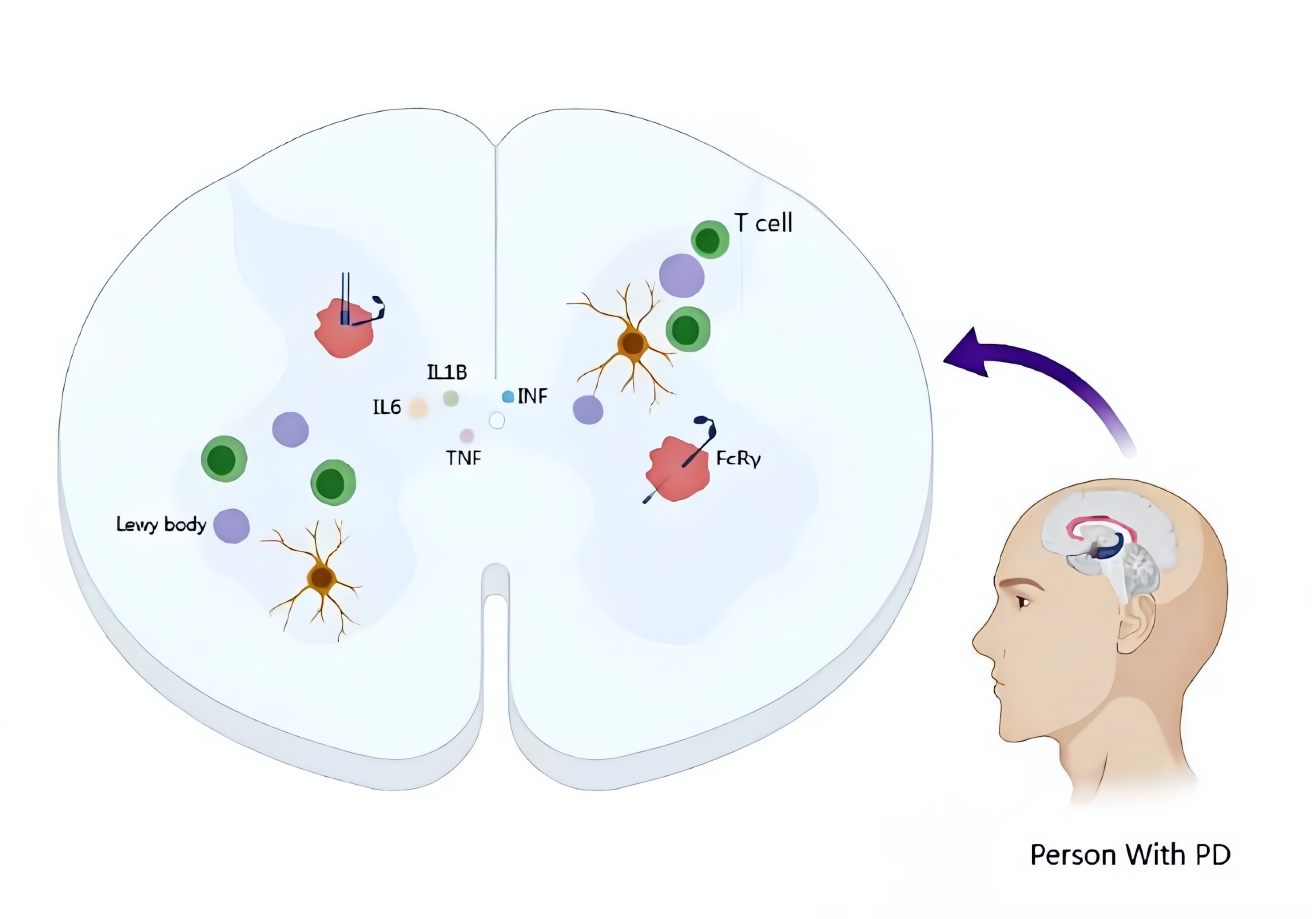
**Pathological features of Parkinson’s disease and the role of neuroinflammation**. Parkinson’s disease (PD) is characterized by the loss of dopaminergic neurons and the production of Lewy bodies. Neuroinflammation arises from the activity of various pro-inflammatory factors. Image created using PPTX (Windows 12.1.0.17133; WPS Office; Beijing, China). IL, interleukin; TNF, tumor necrosis factor; INF, interferon; FcRγ, derived from common γ subunit of Fc receptors.

In addition to microglia and astrocytes, other factors such as gut microbiota and monocyte infiltration are increasingly recognized as contributors to PD-related neuroinflammation [23, 24]. The pathological mechanisms underlying neuroinflammation in PD are complex and involve multiple cellular interactions and signaling cascades, warranting further exploration. Currently, PD diagnosis is predominantly symptom-based, with clinical manifestations often appearing in the late stages of the disease, making early diagnosis challenging [25]. The mechanism of neuroinflammation associated with PD is illustrated in Fig. 1. Given the role of neuroinflammation throughout PD progression, identifying specific inflammatory markers offers promising potential for early diagnosis and targeted treatment strategies. Therefore, we systematically reviewed the key inflammatory factors associated with PD to provide insights that could guide clinical practice.

## Potential Inflammatory Markers of Neuroinflammation in Parkinson’s Disease

In recent decades, the prevalence of PD has increased, yet diagnostic methods remain largely symptom-based, with limited advancements. This has prompted research efforts to identify biomarkers that can more clearly distinguish PD subtypes and facilitate timely diagnosis [26]. Previous studies have identified cerebrospinal fluid (CSF) and blood markers such as alpha-synuclein species, lysosomal enzymes, amyloid, and tau proteins as valuable markers [27]. Given the established link between PD-associated neuroinflammation and pro-inflammatory factors, this section explores the potential of these factors as biomarkers for PD neuroinflammation based on recent studies.

### Interleukin-1β (IL-1β)

The interleukin-1 (IL-1) family consists of various pro-inflammatory and anti-inflammatory proteins, among which IL-1β is notable for its role as a prominent mediator of inflammation. Unlike IL-1α, which is primarily localized to the membranes of limited cell types and thus difficult to detect in blood, IL-1β is secreted into body fluids (such as blood), making it more accessible for measurement [28, 29]. Microglia, which are central to the production of PD neuroinflammation, have been shown to increase IL-1β production in response to the abnormal aggregation of α-synuclein (α-Syn). This process involves the activation of the NOD-like receptor thermal protein domain associated protein 3 (NLRP3) inflammasome in microglia, leading to heightened neuroinflammation [30]. Consequently, IL-1β is considered a potential marker of neuroinflammation in PD.

A study by Fleury *et al*. [31] reported significantly elevated IL-1β levels in individuals with early and mid-stage PD compared to healthy controls. Furthermore, research by Li *et al*. [32] identified significant differences in the rs571556428 allele of IL-1β between PD patients and healthy subjects, suggesting a genetic polymorphism of IL-1β associated with PD. Fan *et al*. [33] further demonstrated that plasma IL-1β levels were significantly higher in PD patients and were positively correlated with Hoehn-Yahr (H-Y) stage and Unified Parkinson’s Disease Rating Scale (UPDRS) Part III scores, suggesting that IL-1β could serve as a non-invasive marker for assessing disease severity. However, it is important to note that IL-1β alone may not provide a comprehensive assessment, and future studies should establish reliable clinical prediction models incorporating IL-1β for more accurate evaluations.

### Interleukin-2 (IL-2)

Interleukin (IL)-2 is a key growth and survival factor for antigen-activated T lymphocytes, and it plays a pivotal role in immune modulation. Due to its immunoregulatory properties, IL-2 has been extensively studied as a therapeutic target for cancer and autoimmune diseases [34, 35]. In PD, Miliukhina *et al*. [36] measured plasma cytokine concentrations in patients with Parkinson’s disease, including those with Glucosylceramidase Beta (*GBA*) gene mutation and sporadic PD, as well as in healthy volunteers using enzyme-linked immunosorbent assay (ELISA) and multiple assays. Their findings revealed elevated IL-2 levels in PD patients [36]. Additionally, a bidirectional Mendelian randomization study by Xue *et al*. [37] demonstrated an association between IL-2 levels and PD risk (odds ratio (OR): 1.18, 95% confidence interval (CI): 1.01–1.38, *p* = 0.041) . Despite early research dating back to 1996 that reported increased IL-2 levels in the cerebrospinal fluid of adolescent PD patients, effective therapies targeting IL-2 have not yet been developed, unlike the extensive research on IL-2 in cancer treatment. It is anticipated that advancements in molecular engineering, especially protein engineering, may eventually lead to the development of PD therapies centered around IL-2 [38].

### Interleukin-6 (IL-6)

Interleukin-6 (IL-6) is a pleiotropic pro-inflammatory cytokine produced primarily by monocytes and macrophages. Its proper expression is essential for host defense and is tightly regulated at multiple levels, including chromatin structure, transcriptional control, and post-transcriptional modifications [39]. Due to the numerous factors influencing IL-6 expression, its trans-signaling mechanism can activate various signaling pathways, including Janus kinase/Signal transducer and activator of transcription (JAK/STAT3) and Phosphatidylinositol 3-kinase and protein kinase B/Akt (PI3K-PKB/Akt), leading to the development of conditions such as cancer, multiple sclerosis, and Alzheimer’s disease [40].

In a study by Jolanta Kwiatek-Majkusiak *et al*. [41], serum levels of ferrimodulin and IL-6 were evaluated in PD patients, revealing significantly elevated levels of both markers and a positive correlation between them. These findings suggest a close link between neuroinflammation and iron metabolism in PD [41]. High IL-6 expression has also been associated with PD prognosis. For example, a study by Veselý *et al*. [42] showed that patients with elevated baseline IL-6 levels exhibited lower depression scores after two years. Similarly, Green *et al*. [43] demonstrated that IL-6 levels are associated with non-motor symptoms in PD patients. The elevated IL-6 levels likely indicate a state of heightened neuroinflammation, which, if sustained, can lead to oxidative stress in the endoplasmic reticulum of microglia. This stress triggers a microglia-dependent immune response, ultimately exacerbating PD progression [44].

Given the dual role of IL-6 in protection and damage, its precise impact on PD neuroinflammation remains unclear. Further research is needed to determine the specific thresholds at which IL-6 contributes to protective or detrimental outcomes in PD patients.

### Tumor Necrosis Factor α (TNF-α)

Tumor necrosis factor α (TNF-α) is a multifunctional cytokine produced by macrophages, monocytes, and neutrophils. It plays a central role in inflammation, apoptosis, and immune system regulation. TNF-α has various biological functions, including resisting infections and contributing to specific pathological conditions [45]. In patients with neuroinflammation, microglia/macrophages may inactivate the vitamin D receptor (VDR), leading to a pro-inflammatory phenotype with elevated TNF-α secretion. This, in turn, enhances endothelial release of CXCL10, disrupts the blood-brain barrier, and promotes peripheral T lymphocyte infiltration [46].

Xiromerisiou *et al*. [47] found that TNF-α levels in PD patients correlated with clinical symptoms, particularly disease severity and cognitive decline. They observed higher TNF-α levels in PD patients at advanced Hoehn-Yahr stages compared to those in early stages, with a significant correlation between TNF-α levels and UPDRS scores. Additionally, Wang *et al*. [48] reported that PD patients with fatigue symptoms were older, had a longer course of disease, and had higher levels of plasma inflammatory cytokines, including IL-1β, IL-18, and TNF-α. Their receiver operating characteristic (ROC) curve analysis showed that TNF-α could be a potential marker for PD-related fatigue, with an AUC of 0.663 and sensitivity and specificity values of 65.71% and 67.86%. These findings underscore the importance of TNF-α as a supplementary marker for predicting symptom severity in PD, complementing current symptom-based diagnostic models.

### Interferon Gamma (IFNγ)

Interferon Gamma (IFNγ) belongs to the Type II IFN family and is primarily produced by immune system cells, including lymphocytes (e.g., natural killer cell (NK), Innate lymphoid cells (ILC)) and adaptive immune cells (e.g., Th1 cell (TH1), Cytotoxic T lymphocyte (CTL)) [49]. As a pleiotropic cytokine, IFNγ signals through its receptors (IFNγR, IFNγR1, and IFNγR2) to mediate various immune responses [50].

Previous studies have highlighted the unique role of IFNγ in microglial activation. IFNγ can induce microglial proliferation, enhance synaptic elimination, and increase nitric oxide release, impairing synaptic transmission and cognitive function. IFN-γ is critical for driving Toll-like receptor (TLR)—activated microglia to neurotoxic phenotypes that induce energy and oxidative stress, severe network dysfunction, and neuronal death [51]. This may be related to Parkinson’s neuroinflammation. However, as a pleiotropic molecule, it is also associated with the differentiation of neural stem cells/progenitor cells (NSPCS), the only pluripotent cell population in the central nervous system, which is involved in the differentiation of various nerve cells. Appropriate levels of IFNγ are believed to have a positive effect on NSPCS differentiation [52]. The specific role of IFNγ in PD remains unclear, but an interesting study by Diaz *et al*. [53] suggested that low IFNγ levels are associated with severe tremors in PD patients, suggesting a potential protective role for IFNγ.

## Treatment

Neuroinflammation plays a pivotal role in the onset and progression of Parkinson’s disease (PD). As previously discussed, various pro-inflammatory factors are upregulated in PD, suggesting that anti-inflammatory therapy could be a key component in managing the disease. This section reviews the impact of non-steroidal anti-inflammatory drugs (NSAIDs), diet, and exercise on PD.

### Non-steroidal Anti-inflammatory Drugs (NSAIDs)

NSAIDs are a class of compounds with anti-inflammatory properties commonly used to treat pain and fever [54]. A 2011 systematic review and meta-analysis by Rees *et al*. [55] revealed that exposure to NSAIDs or aspirin did not significantly affect the risk of developing Parkinson’s disease across 14 studies. However, non-aspirin NSAIDs were associated with a 13% reduction in PD risk, with ibuprofen specifically reducing the risk by 27%, suggesting a potential neuroprotective effect. An *in vitro* study by Dassati *et al*. [56] showed that the selective cyclooxysynthase-2 inhibitor celecoxib (CXB) exhibited direct neuroprotective effects in 6-hydroxydopamine (6-OHDA) and paraquat PD models. Additionally, a bidirectional Mendelian randomization study of 23 drugs conducted by Xie *et al*. [57] found a negative causal relationship between salicylate use and PD, suggesting that salicylate drugs may reduce PD risk. While these studies suggest a protective role for NSAIDs in PD, the translation of these effects into effective treatment remains uncertain, warranting further investigation through large randomized controlled trials.

### Diet

Research has indicated that specific dietary patterns, such as the ketogenic diet and the Mediterranean diet, may benefit PD patients [58]. Growing evidence also links diet to the inflammatory response, making it a relevant consideration for managing inflammation in PD [59]. The ketogenic diet, characterized by low carbohydrate, high fat, and adequate protein intake, mimics fasting by inducing the production of ketone bodies (KB) and promoting a state of ketosis, which can influence neuroinflammation [60]. A study by Jiang *et al*. [61] showed that a 3-month ketogenic diet alleviated cognitive dysfunction and neuroinflammation in APP/PS1 mice via the the nuclear factor erythroid 2-related factor 2/heme oxygenase-1 (Nrf2/HO-1) and nuclear factor kappa-B (NF-κB) signaling pathways. Similarly, a randomized controlled trial by Phillips *et al*. [62] demonstrated that an 8-week ketogenic diet or a low-fat diet improved clinical symptoms in PD patients, with the ketogenic diet being more effective in alleviating non-motor symptoms. These findings underscore the potential of dietary interventions in PD management and warrant further exploration.

### Exercise

Exercise is widely recognized as a beneficial approach for mitigating neuroinflammation. A study by Lianwei Mu *et al*. [63] found that 12 weeks of treadmill exercise inhibited glycogen synthase kinase-3β (GSK3β) kinase activity, reduced levels of amyloid-beta (Aβ) oligomers, decreased pro-inflammatory cytokines (IL-1β, IL-6, and TNF-α), and reduced microglia and astrocytes activation in mice. Additionally, a randomized controlled trial involving 130 PD patients by Johansson *et al*. [64] revealed that aerobic exercise stabilized disease progression in the corticostriatal sensorimotor network and enhanced cognitive performance. These findings highlight the significance of exercise in managing PD, especially in reducing neuroinflammation and supporting cognitive function.

## Conclusion

Neuroinflammation in Parkinson’s disease (PD) involves complex pathological mechanisms, where brain injury triggers the release of pro-inflammatory factors that, in turn, exacerbate neuronal damage. This study highlights potential biomarkers centered on these pro-inflammatory factors, all of which are related to the degree of symptoms of PD to a certain extent. Although extensive research has established a close relationship between these biomarkers and PD, therapeutic interventions targeting them are still lacking. While non-steroidal anti-inflammatory drugs, diet, and exercise have shown promise in mitigating neuroinflammation in PD patients, the evidence supporting their efficacy is still insufficient.

Moreover, although current biomarkers are useful in guiding treatment, they generally lack the specificity required for precise therapeutic interventions. The reliance on traditional treatment approaches underscores the need for innovative therapeutic strategies. Future technological advances are anticipated to deepen our understanding of neuroinflammation and pave the way for large-scale prospective trials to explore the clinical utility of pro-inflammatory factors in predicting and treating PD. Such progress could provide robust evidence to improve the prognosis for PD patients.

## Availability of Data and Materials

Not applicable.
